# Cigarette smoke and nicotine effect on human mesenchymal stromal cell wound healing and osteogenic differentiation capacity

**DOI:** 10.18332/tid/185281

**Published:** 2024-03-16

**Authors:** Janne Heikkinen, Tarja Tanner, Ulrich Bergmann, Sanna Palosaari, Petri Lehenkari

**Affiliations:** 1 Research Unit of Translational Medicine, Faculty of Medicine, University of Oulu, Oulu, Finland; 2 Research Unit of Oral Health Sciences, University of Oulu, Oulu, Finland; 3 Dental Training Clinic, Oulu, Finland; 4 Proteomics and Protein Analysis, Biocenter Oulu, University of Oulu, Oulu, Finland; 5 Medical Research Center Oulu, Oulu University Hospital and University of Oulu, Oulu, Finland; 6 Division of Orthopedic Surgery, Oulu University Hospital, Oulu, Finland

**Keywords:** mesenchymal stromal cells, bone healing and regeneration, cigarette smoke, nicotine, osteogenic differentiation

## Abstract

**INTRODUCTION:**

Mesenchymal stromal cells (MSCs) play a crucial role in promoting tissue regeneration and healing, particularly in bone tissue. Both smoking and nicotine use are known to delay and inhibit the healing process in patients. This study aims at delineating these cellular effects by comparing the impact of nicotine alone to cigarette smoke with equivalent nicotine content, and shedding light on potential differences in the healing process.

**METHODS:**

We examined how cigarette smoke and nicotine affect the migration, proliferation, and osteogenic differentiation of human patient-derived MSCs *in vitro*, as well as the secretion of cytokines IL-6 and IL-8. We measured nicotine concentration of the cigarette smoke extract (CSE) to clarify the role of the nicotine in the effect of the cigarette smoke.

**RESULTS:**

MSCs exposed to nicotine-concentration-standardized CSE exhibited impaired wound healing capability, and at high concentrations, increased cell death. At lower concentrations, CSE dose-dependently impaired migration, proliferation, and osteogenic differentiation, and increased IL-8 secretion. Nicotine impaired proliferation and decreased PINP secretion. While there was a trend for elevated IL-6 levels by nicotine in undifferentiated MSCs, these changes were not statistically significant. Exposure of MSCs to equivalent concentrations of nicotine consistently elicited stronger responses by CSE and had a more pronounced effect on all studied parameters. Our results suggest that the direct effect of cigarette smoke on MSCs contributes to impaired MSC function, that adds to the nicotine effects.

**CONCLUSIONS:**

Cigarette smoke extract reduced the migration, proliferation, and osteogenic differentiation in MSCs *in vitro*, while nicotine alone reduced proliferation. Cigarette smoke impairs the osteogenic and regenerative ability of MSCs in a direct cytotoxic manner. Cytotoxic effect of nicotine alone impairs regenerative ability of MSCs, but it only partly explains cytotoxic effects of cigarette smoke. Direct effect of cigarette smoke, and partly nicotine, on MSCs could contribute to the smoking-related negative impact on long-term bone health, especially in bone healing.

## INTRODUCTION

Smoking poses a substantial public health threat, unequivocally linked to a range of cancers, chronic diseases, and bone health complications. With 1.1 billion individuals using tobacco globally and an annual toll of 7.7 million smoking-related deaths^1^, the ethical exploration of these associations relies on epidemiological studies, autopsies, and diverse scientific models, including perspectives from both animal studies and cellular research. Cigarette smoke, comprising over 7000 chemicals^2^, presents a complex challenge, as the precise agents accountable for adverse health effects and the intricate synergistic toxic mechanisms, remain elusive.

Unravelling the cellular effects of smoking is complicated because many effects manifest themselves only after prolonged low-grade exposure and are challenging to replicate experimentally. Smoking emerges as a risk factor for osteoporosis, increased fracture risk and delayed fracture healing^3^. Nicotine, a prominent compound in cigarettes, while not carcinogenic, induces chemical addiction^4^ and altered tissue physiology, especially vascular regulation. At the physiological level, nicotine initiates vasoconstriction, influencing tissue physiology by altering oxygen levels and pH, yet its precise cell biological effects, especially concerning tobacco smoke, remain unclear. Clarifying these effects gains significance amidst the rising popularity of tobacco-free nicotine products, like nicotine pouches, which are marketed as ‘safer alternatives’ to tobacco smoking^5^.

Studies on the effects of nicotine in bone healing through animal models have yielded conflicting results, showing both negative effects^6,7^ or no effect^8,9^. Mesenchymal stem cells (MSCs), integral to bone healing and regeneration, contribute via immunomodulation and osteoblast differentiation^10,11^. These cells migrate to fracture sites, proliferate, differentiate into osteoblasts, and mineralize the new bone matrix. Their immunoregulatory functions involve interactions with immune cells through adhesion molecules and soluble factors^10^, resulting in the secretion of cytokines like TNF-α, TGF-B, IL6, and IL8^12^. IL6 and IL8 are particularly intriguing due to their roles in bone remodeling, inflammation, and associations with chronic autoimmune diseases^11,13^. Prolonged elevation of IL6 and IL8, seen in rheumatoid arthritis, chronic obstructive pulmonary disease, and type II diabetes, underscores their potential deleterious effects^14-16^, all of which are known to be influenced by smoking^17^.

While the detrimental effects of tobacco smoke on bone health are well-established in patients, the direct negative effects of nicotine on osteogenic differentiation and bone metabolism need to be studied further at cellular level. This study aims at delineating these cellular effects by comparing the impact of nicotine alone to cigarette smoke with equivalent nicotine content, and shedding light on potential differences in the healing process.

## METHODS

### Human MSCs isolation and culture

Bone marrow samples for MSC isolation were collected with the approval of the institutional Ethics Committee of the Northern Ostrobothnia Hospital district, Finland. Written informed consent was obtained from each patient and the procedures followed the principles of the Helsinki Declaration. Three primary human cell samples were used in this study from patients (a male aged 44 years, a male aged 41 years, and a female aged 72 years) with post-traumatic or primary hip osteoarthritis, without any known condition that could influence the bone formation capacity.

The cells used in this study are described as mesenchymal stromal cells instead of mesenchymal stem cells, since the expression of surface markers as defined by Dominici et al.^18^ was not tested. The cells were bone marrow derived, plastic-adherent cells with a confirmed potential to adipogenic and osteogenic differentiation. Isolation and culture of MSCs were performed as described previously^19^. Briefly, samples were collected from patients undergoing hip replacement surgery. Bone marrow was obtained from the femoral shaft and trochanteric region during the operation. The bone marrow sample was transferred to a T-175 cell culture flask (Greiner, Germany) containing basal medium. After a few days unattached cells were washed away and adherent cells were used in experiments. Cells of the donors were randomly selected. Primary cells that were sub-cultured for maximum of 7 passages were used in the experiments for securing proliferation and differentiation capacity and avoiding cell senescence. All experiments were repeated three times.

Cells were cultured in basic Alpha Medium (Corning, New York, USA) with 10% fetal bovine serum (Biowest, Riverside, MO, USA), 20 mM HEPES (Corning, New York, USA) and 1% penicillin-streptomycin (Gibco, Waltham, MA, USA). For osteogenic differentiation 100 nM dexamethasone (Sigma-Aldrich, Saint Louis, MO, USA), 50 mg/mL ascorbic acid (Sigma-Aldrich, Saint Louis, MO, USA) and 10 mM beta-glycerolphosphate (Sigma-Aldrich, Saint Louis, MO, USA) were added. Cellular experiments were conducted with cells of the three donors, with six replicates on 96-well plates (Greiner, Kremsmünster, Austria) with a cell density of 1500 cells per well. In the scratch-wound assays, the cell density was 10000 cells per well.

### Cigarette smoke extract preparation

Cigarette smoke extract (CSE) preparation was performed as described previously^20^. Briefly, the mainstream smoke of two cigarettes (‘North State’) without a filter was bubbled through 40 mL of culturing medium with an evacuation ejector (AGA MS-33, Solna, Sweden). The bubbling and burning time of one cigarette was four minutes. The solution was sterilized by filtration through a 0.2 μm filter and kept at -80°C in 1 mL aliquots until use. For each medium change, CSE was diluted in basal or osteogenic medium. Media was changed twice a week during the whole experiment. The exposure media contained 50, 100 and 500 ng/mL of nicotine in CSE and the same concentrations were used for plain nicotine (EMD Millipore, Burlington, MA, USA). A nicotine stock of 0.5 mM was made in culture medium and the stock was kept at -80°C in 1 mL aliquots until use. For each medium change nicotine was diluted in basal or osteogenic medium.

### Quantification of nicotine by mass spectrometry

Nicotine was quantified from CSE by accurate mass measurement using LCMS with a synapt G2 Q-ToF mass spectrometer coupled to an Aquity chromatography system (Waters) using a method based on the work of McGuffrey et al.^21^. The mass spectrometer was operated at 2 Hz in positive mode in the m/z range of 100 to 600 with lock mass (Leu-enkephalin) correction. The chromatography column (Waters Aquity BEH C18 2.1×150 mm) was eluted at 0.35 mL/min with a linear gradient from 99% A (10 mM ammonium acetate) to 90% B (methanol) in 9 min. Quantification was done with the Quanlynx option of Masslynx (Waters), using SIC with accurate mass of the candidate compounds.

### Migration assay

In three independent experiments, a total of 132 scratch wounds were analyzed for cell motility and proliferation under nicotine or CSE exposure, using IncuCyte S3 Live-Cell imaging system (Essen BioScience, Sartorius) and Incucyte 2021 C software. All experiments were performed as 4 technical replicates per condition (12 wells per group). The wound area was imaged every two hours for 50 hours and the healing was analyzed as the wound width and confluence (area %) of the wound area.

### Metabolic activity and cell viability

Metabolic activity of MSCs was assessed by MTT-tests (3-[4,5-Dimethylthiazol-2-yl]-2,5-diphenyltetrazolium bromide, Methyl Thiazolyl Tetrazolium) as described previously^22^ , using cells from three donors. All experiments were performed in 3 biological and 6 technical replicates (18 wells per group) and a total of 864 wells were analyzed. The metabolic activity was analyzed after 24 hours, 21 days and 35 days of nicotine or CSE exposure. At each time point the cells were incubated 4 hours with 100 μL medium containing 0.5 mg/mL MTT-substrate. MTT-medium was removed, and the cells lysed with 100 μL DMSO (Fisher Scientific, Waltham, MA, USA) to release the formazan dye from the cells. Absorbance of each well was quantified with a plate reader (Victor 2, PerkinElmer Life Science/Wallac Oy, Turku, Finland) at 550 and 650 nm, and the background at 650 nm was subtracted from the absorbance at 550 nm. The results were normalized to controls at 24 h.

### Alkaline phosphatase activity

The increase in alkaline phosphatase (ALP) activity and calcium deposition in the extracellular matrix are markers of osteogenic differentiation. After 21 days of osteogenic differentiation with or without exposure to nicotine or CSE, the alkaline phosphatase (ALP) activity of MSCs was measured using 4-nitrophenyl phosphate as described previously^19^. The protein contents of the samples were determined by Bio-Rad Protein Assay (Bio-Rad Laboratories, Richmond, CA) and ALP activity was expressed as arbitrary units relative to protein content (units/mg). Cells from the three donors (total of 144 wells) were used in the experiments in 6 technical replicates (18 samples per group).

### Calcium deposition

Calcium deposition in the extracellular matrix was measured after 35 days of differentiation and exposure using a Calcium Assay Kit (Abcam, Cambridge, UK) according to the manufacturer’s instructions as described previously^19^. Cells from the three donors (total of 144 wells) were used in the experiments in 6 technical replicates (18 samples per group).

### PINP and PIIIP ELISA

Type I collagen is a marker for bone formation and type III collagen is produced in wound healing and as a response to cell stress. N-Propeptide of Type I Procollagen (PINP) and N-Propeptide of Type III Procollagen (PIIINP) were analyzed from the cell culture medium after 21 days of differentiation using Aviva systems biology (San Diego, CA, USA) PINP and PIIINP ELISA Kits according to the manufacturer’s instructions. Due to limited sample volume, 3 technical replicates from each donor were pooled and analyzed as duplicates. A total of 96 samples were analyzed for PINP and PIIINP presenting 3 biological and 6 technical replicates per group. The CV% for all technical replicates in the ELISA measurements was <10%.

### IL6 and IL8 ELISA

IL6 and IL8 were analyzed from the medium after 21 days of differentiation using Invitrogen (Waltham, MA, USA) IL6 and IL8 ELISA kits according to the manufacturer’s instructions. The CV% for all technical replicates in the ELISA measurements was <10%. Sample preparation, analysis and reporting were similar to those described in the PINP and PIIINP.

### Statistical analysis

SPSS statistics software, version 25 (IBM corp. Armonk, NY, USA) was used for statistical analyses. The Kruskal-Wallis test was used to investigate statistically significant differences between the test groups, and Mann-Whitney U-test was used for pairwise comparison. The direction and strength of the dose responses were analyzed using Spearman’s correlation, and p-values to correlation coefficient (R) were calculated using the t-test. All tests were analyzed as two-tailed, and p<0.05 was considered statistically significant, no adjustments for multiple comparisons were performed.

## RESULTS

### Effects of CSE and nicotine exposures on MSC migration and proliferation

**Figure 1:**
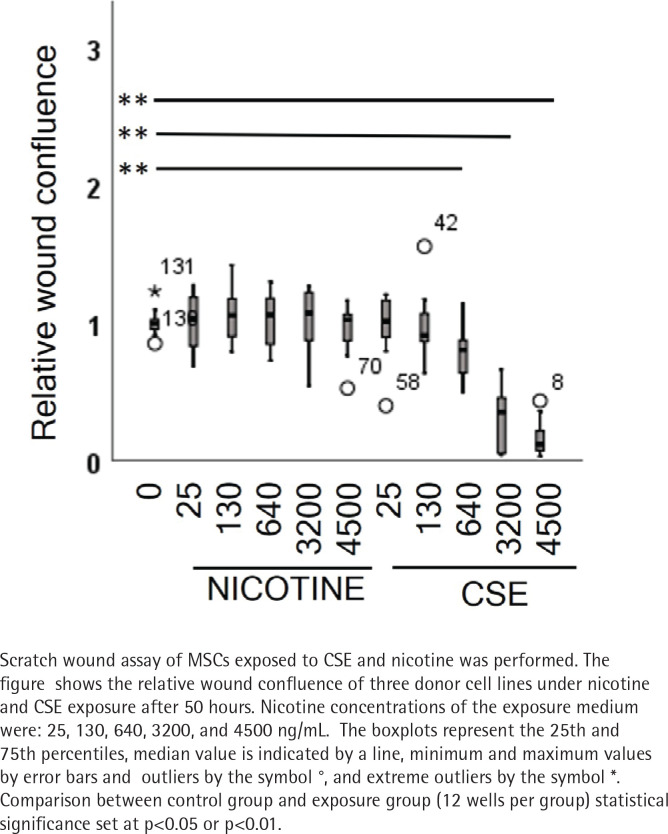
CSE had a strong inhibitory effect on the wound confluence

In the first experiment, we wanted to define the nicotine and CSE concentrations that could be used in the long-term differentiation experiments. The aim was to ensure that the monitored effects during osteogenic differentiation were not caused by cell death but other cellular responses. For this, 132 scratch wounds were analyzed to study the effects of CSE and nicotine on cell migration and proliferation with a wide range of nicotine concentrations (0 to 4500 ng/mL). In the control group (n=12), wound closure occurred within 2 days. Nicotine exposure did not result in any dose-dependent differences in wound closure or cell confluence when compared to the control group (Figure 1, and Supplementary file Figures 1–4).

However, in cells exposed to CSE, we could see a dose-dependent delay in the wound closure. This was shown as positive correlation between the wound width and CSE dose (r_s_=0.512, p<0.001) and conversely, as a negative correlation of the CSE dose with the confluence of cells within the wounds (r_s_= -0.447, p=0.010) (Figure 1, and Supplementary file Figure 4). The two highest CSE concentrations (3200 and 4500 ng/mL nicotine) exhibited toxic responses including cell shrinkage and detachment leading to cell death (Supplementary file Figure 5). As a consequence, these two groups were excluded from the statistical analyses but are represented with dotted lines in Supplementary file Figures 2 and 3.

Next, we employed an MTT assay to assess the impact of long-term exposure to nicotine and CSE on metabolic activity during osteogenic differentiation and in basal medium. In these experiments we selected the exposure concentrations (0–500 ng/mL) that left the cells viable in the wound healing assays. After 21 days of exposure in basal medium, both nicotine (r_s_= -0.327, p=0.002) and CSE (r_s_= -0.497, p<0.001) showed a dose-dependent decrease in the metabolic activity (Supplementary file Figure 6). In osteogenic conditions, nicotine (r_s_= -0.227, p=0.03) but not CSE (r_s_= -0.146, p=0.220) exposure caused a statistically significant change in the metabolic activity, although we could see some changes in the activity under CSE exposure (Supplementary file Figure 7).

### Effects of CSE and nicotine exposures on MSC osteogenic differentiation

**Figure 2:**
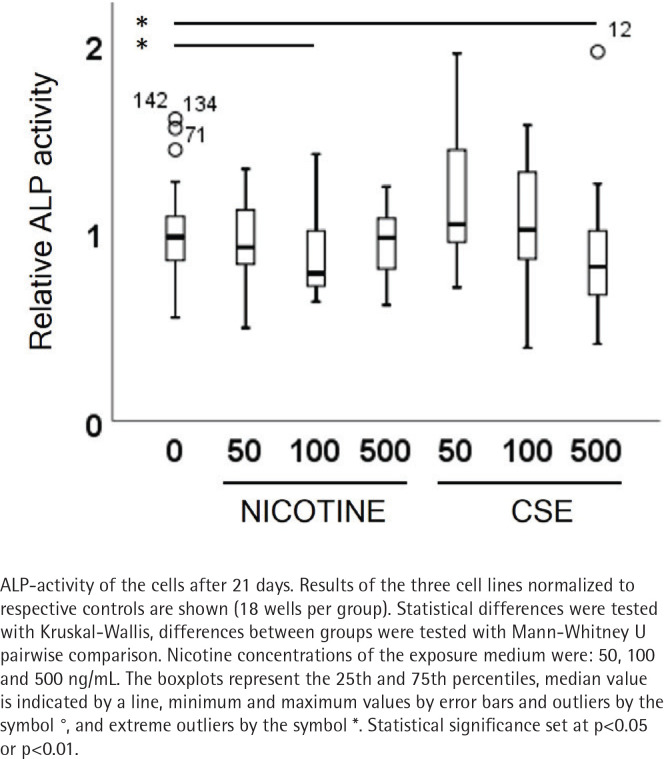
The CSE inhibited ALP activity during osteogenic differentiation

**Figure 3:**
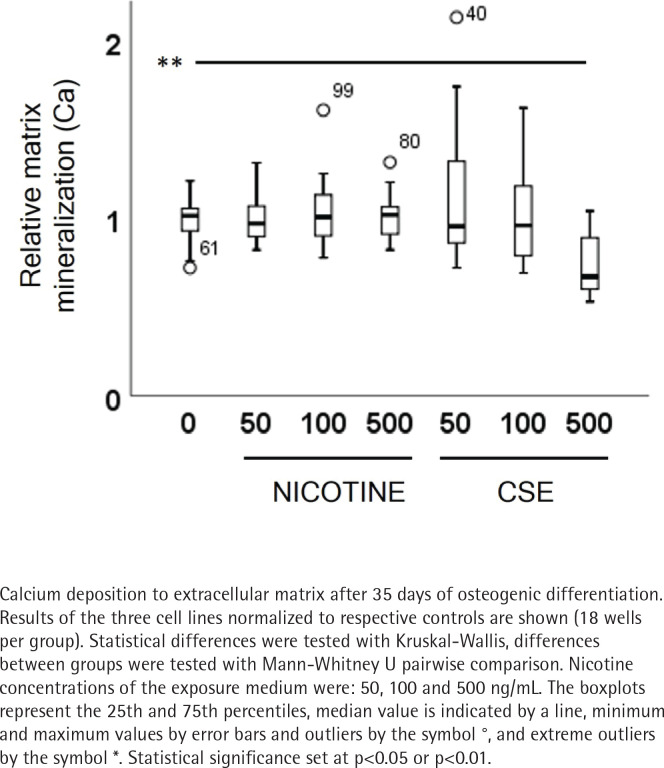
The CSE inhibited matrix mineralization of MSCs during osteogenic differentiation

ALP activity and calcium deposition in extracellular matrix were measured to observe the nicotine and CSE exposure effects on osteogenic differentiation of the MSCs. After 21 days of osteogenic differentiation, ALP activity did not show any change in the nicotine exposed cells, while there was a slight increase in ALP activity in the low (50 ng/mL) CSE exposure and a decrease in ALP activity with the highest (500 ng/mL) CSE exposure, with a statistically significant difference between the highest CSE exposure and the control group (Mann Whitney U, p=0.025) (Figure 2). Also, the calcium deposition after 35 days of exposure was unaffected by the used nicotine concentrations (0–500 ng/mL), but CSE exposure with similar nicotine concentrations showed a dose-dependent decrease in the calcium deposition (r_s_= -0.468, p<0.001) (Figure 3).

**Figure 4:**
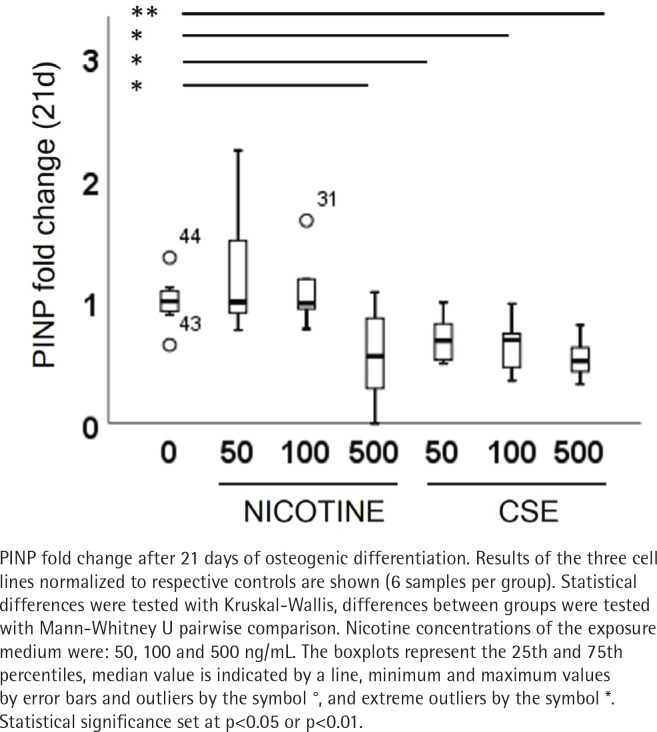
The CSE and nicotine inhibited PINP secretion of MSCs during osteogenic differentiation

Type I and III collagen markers, PINP and PIIINP, were measured from the medium after 21 days of osteogenic differentiation. Both nicotine (r_s_= -0.320, p=0.085) and CSE (r_s_= -0.725, p<0.001) decreased the type I collagen production dose-dependently, but the decrease was statistically significant only with CSE exposure (Figure 4). Highest nicotine exposure (500 ng/mL) lowered PINP secretion in a statistically significant manner compared to the control group (Mann Whitney U, p=0.019) (Figure 4). Type III collagen secretion was low in all conditions and neither nicotine nor CSE exposure had a statistically significant change in the PIIINP concentrations (Supplementary file Figure 8).

### Effects of CSE and nicotine exposures on MSC IL6 and IL8 secretion

MSCs in native state (basal medium) reacted to nicotine exposure with a dose-dependent increase in IL6 secretion, but the change was not statistically significant (r_s_=0.312, p=0.094) (Supplementary file Figure 9). Nicotine caused no effects on IL6 secretion in osteogenic conditions (Supplementary file Figure 10). CSE exposure showed a decreasing trend in IL6 secretion both in basal and osteogenic conditions, but the changes were not statistically significant.

MSCs in basal or osteogenic conditions showed no nicotine induced responses in IL8 secretion, but CSE exposure changed IL8 secretion slightly in basal conditions and increased IL8 secretion significantly in osteogenic conditions (r_s_=0.584, p=0.001) (Supplementary file Figures 11 and 12).

## DISCUSSION

Smoking is one of the main risk factors for impaired bone healing^23^, but the exact cellular mechanism is not fully known. In this study, we specifically addressed the role of mesenchymal stem cells *in vitro*, while bone healing at the cellular level *in vivo* is a complex and lengthy process involving the influx of inflammatory cells in the acute phase, followed by the activation of mesenchymal cells, as reviewed by Marsell and Einhorn^11^, which serve as the effector cells in connective tissue regeneration and healing. Hence, all data presented here should be interpreted as a section of the whole process.

MSCs are the progenitors of osteoblasts, which, over time, differentiate into osteocytes, and the function of these cells is detrimental to bone health. In experimental studies on the effects of cigarette smoke extract, the exposure media have been prepared using standard conditions^24-26^, but mostly without standardization of the extract constituents. In the current study, we measured the nicotine concentration in CSE and compared the effects of CSE and nicotine only on the osteogenic differentiation of human MSCs to clarify the relationship between tobacco smoke and nicotine behind the adverse effects of smoking on bone health during MSC differentiation.

In this study, we showed that CSE has a direct cytotoxic effect on MSCs that is not fully explained by the nicotine effect. At viable exposure concentrations, CSE interferes with osteogenic differentiation with a dose-dependent decrease in MSC migration and proliferation, all of which are required for optimal bone healing, also *in vivo*.

### CSE and nicotine have an adverse effect on MSC’s healing capacity

In this study, we intentionally used a range of nicotine concentrations from 50 to 500 ng/mL. Based on earlier *in vivo* studies, nicotine concentrations in smokers’ blood vary from 4 to 72 ng/mL^27^, but there is high inter-individual variation in blood nicotine concentration, even after the same number of cigarettes. Blood nicotine concentration can rise up to 100 ng/mL^28^, and there are very few exact data on the highest concentrations directly after the smoking event. Based on this information, testing the nicotine effect with concentrations up to 100 ng/mL *in vitro* is likely to be clinically relevant. However, studies on nicotine effects have often relied on concentrations that are much higher than those found in the human body. Kim et al.^29^ showed a decrease in MTT activity over 2 mM nicotine and osteogenic markers on 2 mM (320 μg/mL) nicotine, and both Shaito et al.^25^ and Cyprus et al.^26^ demonstrated the impairment of osteogenic differentiation of human MSCs at high doses of CSE. Because high nicotine and CSE concentrations have been used, the immediate and toxic effects have received more attention than low-grade long-term exposure.

The key steps for MSCs in bone healing are first to migrate and home to the fracture or remodeling area, proliferate, and differentiate. In this study, patient-derived MSCs showed significant wound healing and bone formation capacity. We found that CSE exposure with nicotine concentrations >500 ng/mL has a direct and systematic cytotoxic effect on MSCs that compromises MSC function. Interestingly, the high-dose CSE effect on MSC migration seems to be driven by a complex process of cytoskeleton rearrangement that can be detected as a change in cell morphology. We did not observe this effect with the same concentration of nicotine alone in the short-term experiments, but we could observe the dose-dependent inhibitory effect of nicotine on cellular metabolism already at 50 ng/mL and 100 ng/mL in the long-term exposure (21 days). With CSE at respective nicotine concentrations, we could observe the effect on both MSC migration and proliferation after 2 days and the inhibitory effects were replicated in the 21-day metabolic assays (MTT). At this point, we are not able to suggest any plausible explanation for the CSE cytoskeletal effect, but it is well known that CSE contains many elements that could potentially have a direct effect on microtubules or actin assembly.

### CSE and nicotine have an adverse effect on osteogenic differentiation

In addition to the effects on migration and proliferation, our data on human MSCs clearly show a dose-dependent decrease in the expression of osteogenic markers under long-term CSE exposure. Type I collagen is the main constituent of bone, responsible for bone strength, together with bone minerals. Epidemiological evidence has shown the negative effects of smoking on bone mineral density, risk for osteoporosis, and impaired bone healing^3^. The *in vivo* effect can be multifactorial, but our experimental evidence supports the hypothesis that the bone cells themselves are a plausible key target. Already at a clinically relevant CSE dose (50 ng/mL nicotine), we observed lower calcium deposition and type I collagen production, and this effect became more evident with the increasing CSE dose. ALP activity initially increased with the lowest CSE dose and decreased with the increasing CSE dose. The small increase can be interpreted as a sign of cellular stress. Cigarette smoke-related decrease in MSC proliferation and impaired bone formation, due to the disruption of cellular function, may partially explain poor bone healing and other bone formation-related problems in smokers^3^.

Our experimental conditions are closer to the effects of cigarette smoking in the body than many of the previous studies^25,26^, and one of the strengths of this study is that we measured the nicotine concentration of CSE to be able to compare the CSE effect with nicotine alone. We did not find a statistically significant change in calcium deposition or ALP activity caused by nicotine alone, but there was a decreasing effect on type I collagen production and MSC proliferation. Earlier findings on the effects of low nicotine concentrations have been contradictory. While Kim et al.^29^ did not find cellular effects for nicotine at concentrations below 100 μM, Ng et al.^30^ reported nicotine decreasing osteogenic markers at 1 μM (162 ng/mL) concentration. Our exposure concentrations were in the same range as Ng et al.^30^, and our data agree partly with their findings.

### Cytokine response to CSE and nicotine

IL6 plays a central role in the beginning of fracture healing, where it induces mesenchymal cell proliferation and differentiation^11^. In bone remodeling, IL6 drives the differentiation of osteoclasts and osteoblasts^11^. Unlike our other measurements, IL6 responses showed considerable individual variation in low-dose long-term exposure to CSE or nicotine. This kind of individual variation has also been shown in systemic cytokine production among smokers^31^. In previous studies, the effects on cytokines have been contradictory. CSE has been reported to increase IL6 and IL8 gene expression^26^ but decrease their secretion^24^. However, a 24-h nicotine exposure (0.3 mg/mL) was shown to double IL6 secretion in human umbilical cord MSCs^32^. In our experimental conditions, closer to physiological nicotine levels, the effect is less evident, but it seems that IL6 secretion is not exhausted even under long-term exposure. This could contribute to smoking-related changes in bone healing and remodeling, and the development of inflammatory diseases. The IL6 effect could be mediated through the impact on vasculogenesis^33^, increased and prolonged inflammation, or by IL6-mediated osteolytic effects through osteoblasts and osteoclasts^34^.

In our experiments, MSCs constantly secreted more IL8 in osteogenic medium, and this was further dose-dependently elevated under CSE exposure, but not with nicotine alone. IL8 secretion has been associated with the induction of osteogenic differentiation of MSCs^35^. IL8 is a known activator of osteoclastogenesis, and is thus involved in bone remodeling and osteoblast-osteoclast crosstalk^36^. IL8 also has activating effects on polymorphonuclear cells that can drive the local environment towards inflammation. Systemically, a difference in the cytokine balance can lead to a significant change in the healing process because multiple cytokine-responding cells are always present in the healing granulomatous tissue; hence, these data might not be applicable to interpreting the situation *in vivo*.

Overall, bone healing after trauma is a lengthy process, and in a smoker’s body, continuous exposure to tobacco smoke can delay or even prevent the healing process^3^. Even if the adverse effects on bone healing in clinical settings are partly due to tobacco and nicotine-related changes in circulation and the risk of infections, there are also direct cellular effects on fracture union and bone quality^23^. Thus, we believe that our prolonged experimentation reflects the natural bone healing process that lasts for weeks and could explain some of the direct effects on bone formation. Disturbance in proliferation together with weakened migration can lead to decreased availability of MSCs in the healing tissue, which could potentiate the detrimental effect of smoking *in vivo*.

### Limitations

We consider the low number of cell lines as a limitation of our study. All the CSE inhibitory effects on migration, proliferation, and osteogenic differentiation were shown in all three investigated patient cell lineages. As expected, there was variation between individuals, but the CSE effects seem to be consistent, except for the IL6 responses, which is in line with the clinical findings among smokers^31^. However, our experimental data cannot be directly extrapolated to the events on-going *in vivo*, and our results are based on data reflecting the changes in limited number of parameters on protein level. A proteomics or transcriptomics approach could give a more elaborate view on all the changes induced by CSE or nicotine exposure. Further studies are needed to confirm the clinical significance of this effect.

## CONCLUSIONS

Cigarette smoke extract reduced the migration, proliferation, and osteogenic differentiation in MSCs *in vitro*, while nicotine alone reduced proliferation. Cigarette smoke impairs the osteogenic and regenerative ability of MSCs in a direct cytotoxic manner. Cytotoxic effect of nicotine alone impairs regenerative ability of MSCs, but it only partly explains cytotoxic effects of cigarette smoke. Direct effect of cigarette smoke, and partly nicotine, on MSCs could contribute to the smoking-related negative impact on long-term bone health, especially in bone healing.

## Supplementary Material



## Data Availability

The data supporting this research can be found in the Supplementary file.
