# Supplementary material for: Cigarette smoke and nicotine effect on human mesenchymal stromal cell wound healing and osteogenic differentiation capacity
Source: Tob Induc Dis. 2024 Mar 16;22:10.18332/tid/185281. doi: 10.18332/tid/185281 (PMC10943629; doi:10.18332/tid/185281)
Supplement: Supplementary file 1 [file TID-22-54-s1.pdf]

Cigarette smoke has a direct effect on human mesenchymal stromal cell wound healing and osteogenic differentiation capacity

Janne Heikkinen*, Tarja Tanner, Ulrich Bergmann, Sanna PalosaariΔ and Petri LehenkariΔ

Δwith equal contribution

*Corresponding author

Supplementary file S1

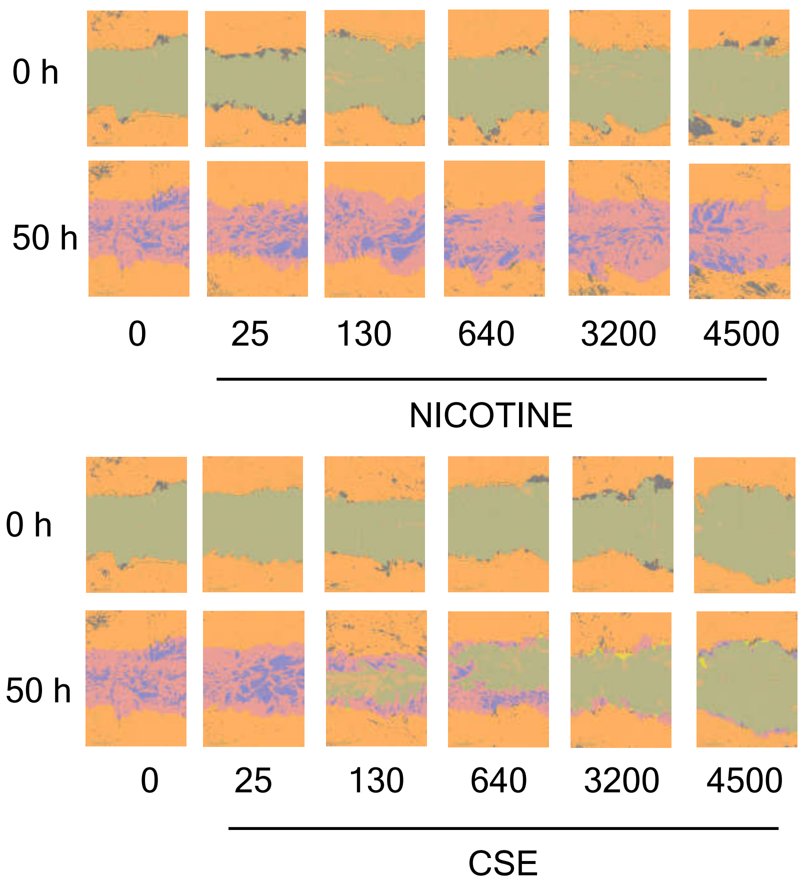

Supplementary figure 1. CSE had a strong inhibitory effect on the wound closure and confluence. Scratch wound assay of MSCs exposed to CSE and nicotine was performed. Figure 1. shows series of exemplary images with cell confluence mask (IncuCyte S3) from scratch wound assay at time points 0 h and 50 h when wounds in the control group were closed.

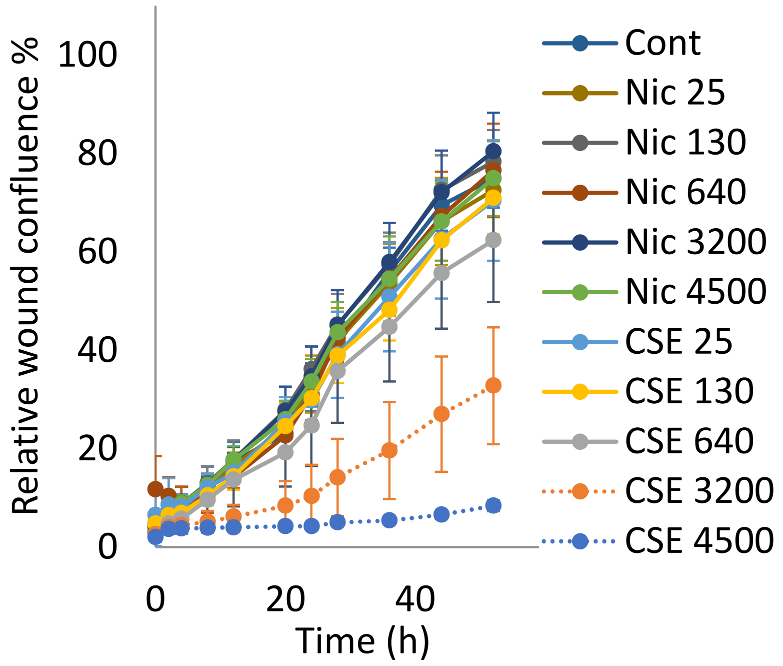

Supplementary figure 2. CSE had a strong inhibitory effect on the wound confluence – a time series. Scratch wound assay of MSCs exposed to CSE and nicotine was performed. Figure 2. shows relative wound confluence during the time-series under CSE and nicotine exposure. 25, 130, 640, 3200 and 4500 (ng/ml) = nicotine concentration of the exposure medium. Data is presented as mean and error bars represent standard deviation.

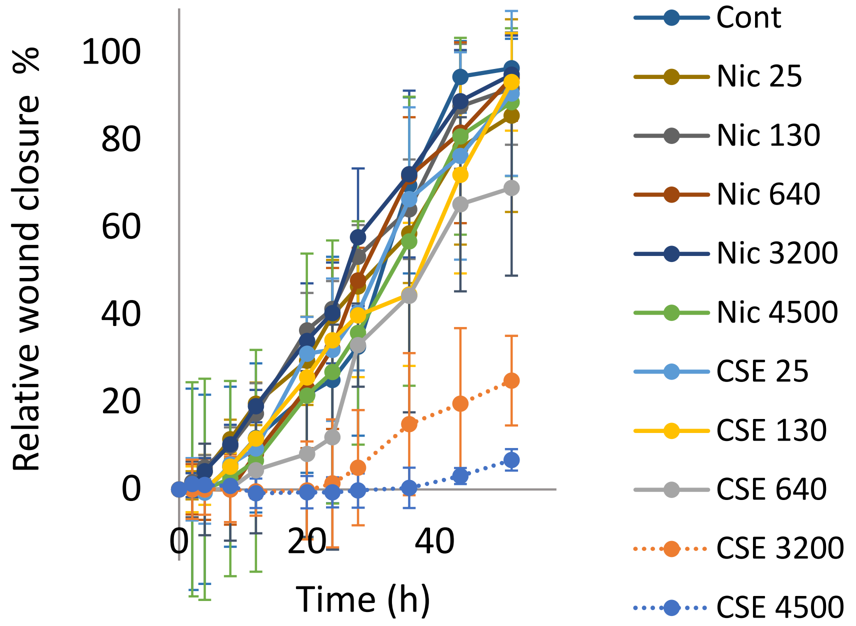

Supplementary figure 3. CSE had a strong inhibitory effect on the wound closure. Scratch wound assay of MSCs exposed to CSE and nicotine was performed. Supplementary figure S2. shows relative wound closure during the time-series under CSE and nicotine exposure. 25, 130, 640, 3200 and 4500 (ng/ml) = nicotine concentration of the exposure medium. Data is presented as mean and bars represent standard deviation.

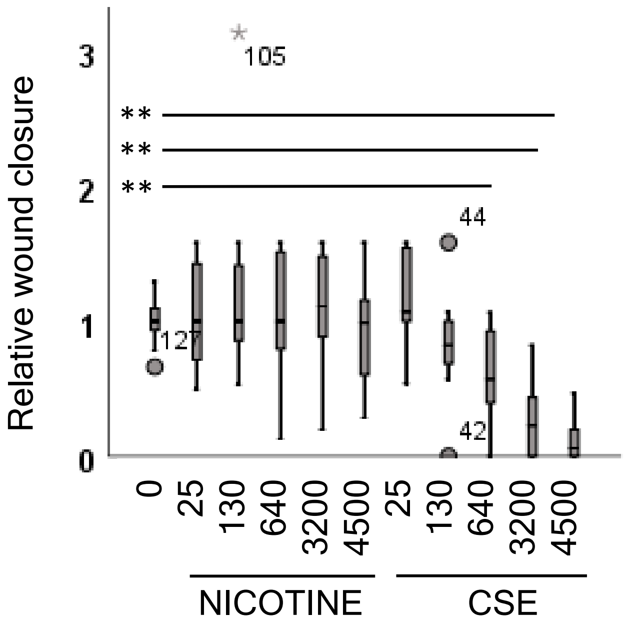

Supplementary figure 4. CSE had a strong inhibitory effect on the wound closure. Scratch wound assay of MSCs exposed to CSE and nicotine was performed. Supplementary figure S3. shows relative wound closureof three donor cell lines under CSE and nicotine exposure after 50 hours. 25, 130, 640, 3200 and 4500 (ng/ml) = nicotine concentration of the exposure medium. **p <0.05 *p<0.01* comparison between control group and exposure group. The boxplots represent the 25th and 75th percentiles, median value is indicated by a line, minimum and maximum values by error bars and ° outliers, * extreme outliers.

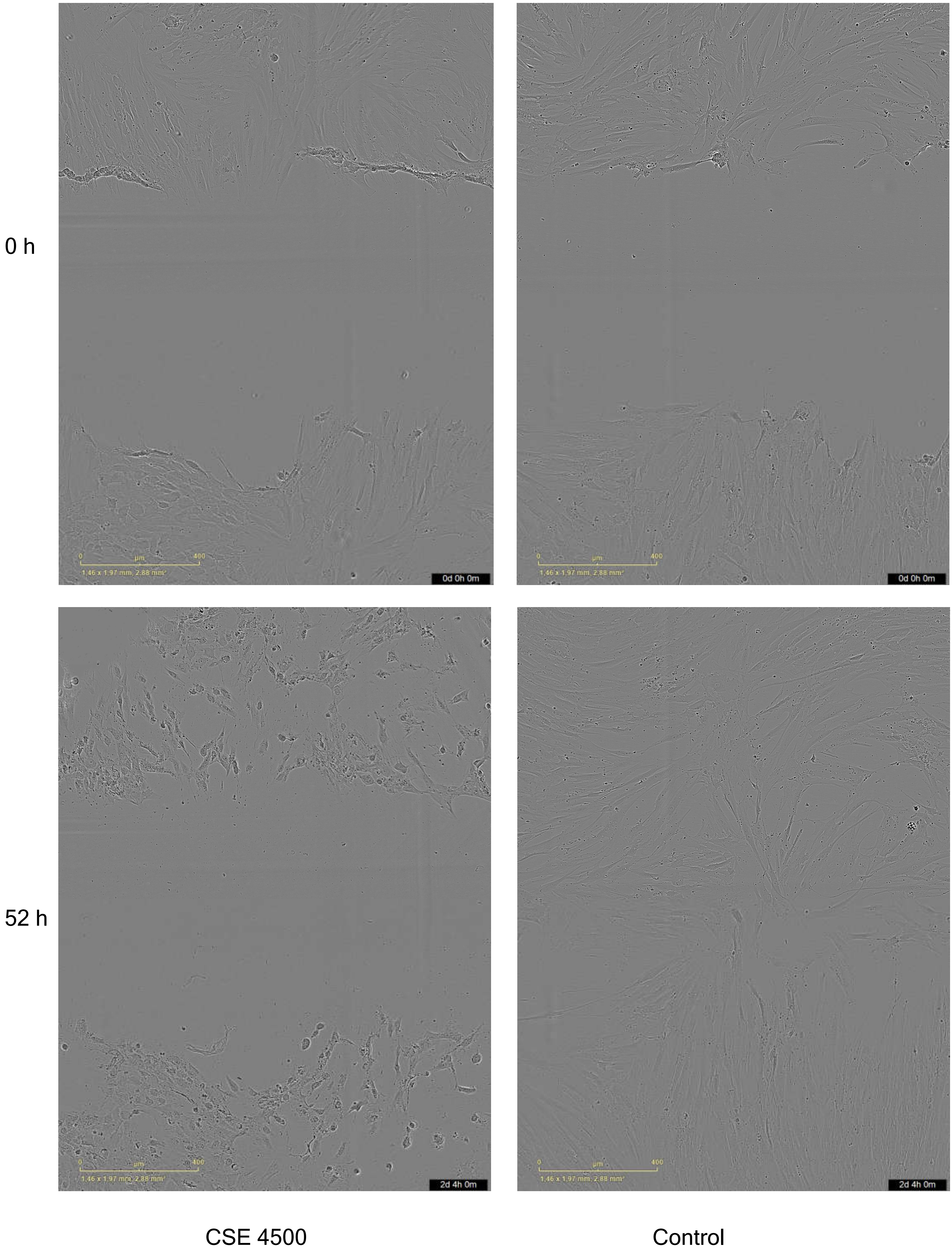

Supplementary Figure 5. Two strongest CSE concentrations caused apoptotic effects.
Microscopic image of cells exposed to strongest CSE (CSE 4500) concentration and control group (0) in time of 0 h and 52 h.

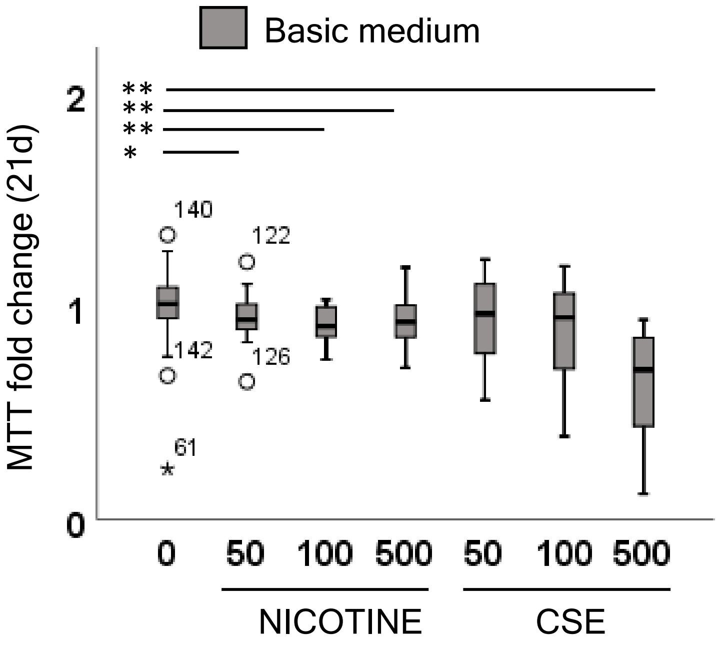

Supplementary figure 6. CSE and nicotine showed dose dependent decrease in metabolic activity in basal medium. Data from MTT-assays is presented as fold change absorbance between MSCs exposed to CSE and nicotine and controls in basal medium after 21 days. Results of the three cell lines normalized to respective controls are shown (18 wells per group). Statistical differences between the groups were tested with Kruskal-Wallis test, pairwise comparison with Mann-Whitney U test. P-values <0.05 were considered statistically significant. * *p<0.05,* ** *p<0.01*; 50, 100 and 500 = nicotine concentration of the exposure medium (ng/ml). The boxplots represent the 25th and 75th percentiles, median value is indicated by a line, minimum and maximum values by error bars and ° outliers, * extreme outliers.

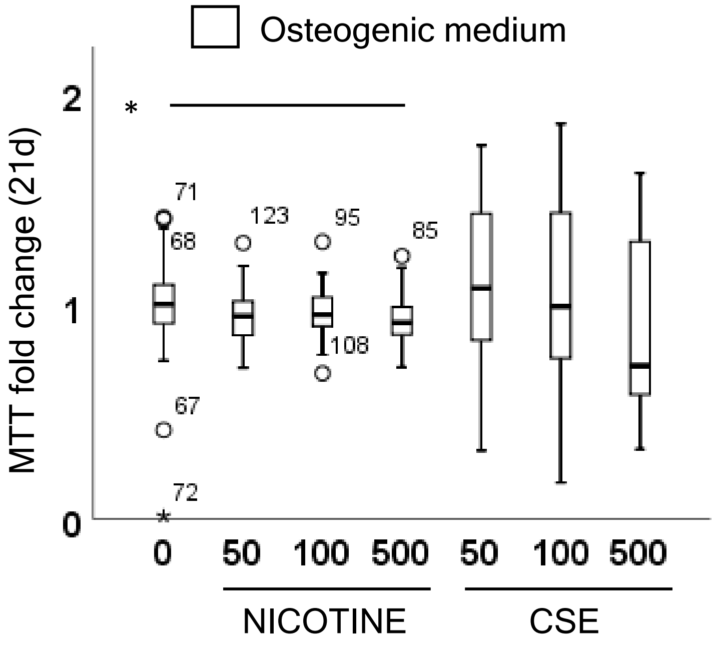

Supplementary figure 7. Effects of CSE and nicotine to metabolic activity in osteogenic condition. Data from MTT-assays is presented as fold change of absorbance between MSCs exposed to CSE and nicotine in osteogenic medium after 21 days. Results of the three cell lines normalized to respective controls are shown (18 wells per group). Statistical differences between the groups were tested with Kruskal-Wallis test, pairwise comparison with Mann-Whitney U test. P-values <0.05 were considered statistically significant. * *p<0.05, ** p<0.01;* 50, 100 and 500 = nicotine concentration of the exposure medium (ng/ml). The boxplots represent the 25th and 75th percentiles, median value is indicated by a line, minimum and maximum values by error bars and ° outliers, * extreme outliers.

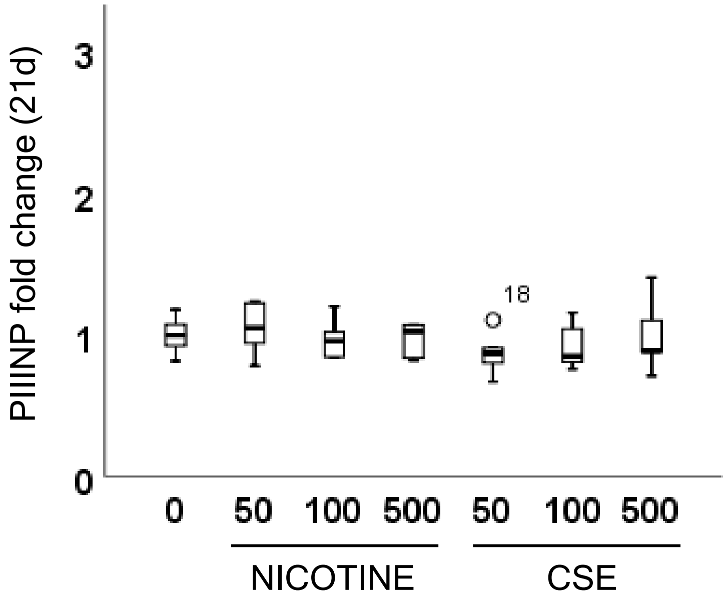

Supplementary figure 8. CSE and nicotine did not affect the secretion of PIIINP. PIIINP secretion to the medium after 21 days of osteogenic differentiation. Results of the three cell lines normalized to respective controls are shown (6 samples per group). Statistical differences were tested with Kruskal-Wallis, differences between groups were tested with Mann-Whitney U pairwise comparison. P-values <0.05 were considered statistically significant. * *p<0.05,* ** *p<0.01* 50, 100 and 500 = nicotine concentration of the exposure medium (ng/ml). The boxplots represent the 25th and 75th percentiles, median value is indicated by a line, minimum and maximum values by error bars and ° outliers, * extreme outliers.

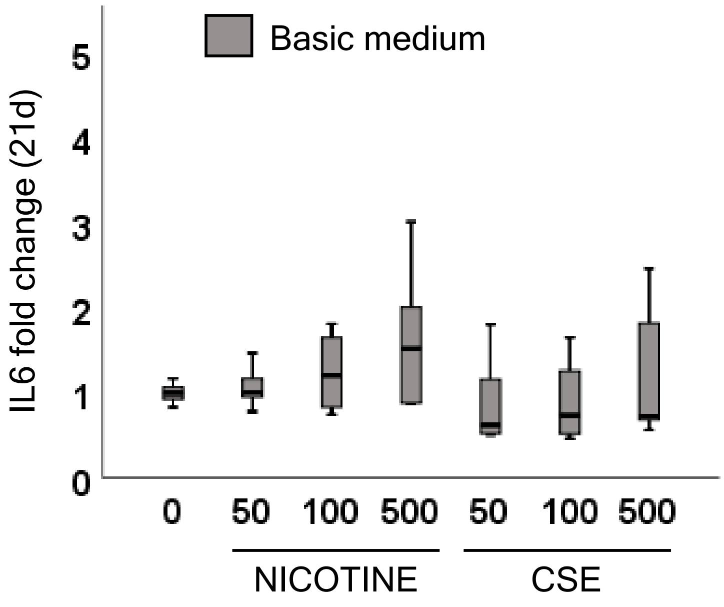

Supplementary figure 9. The effect of CSE and nicotine on cytokine secretion. The cytokine data (ELISA) of IL6 in the medium in basal condition was analysed after 21 days. For the ELISA assay, results of the three cell lines normalized to respective controls are shown. Medium from three biological replicates were combined and the samples were measured as two technical replicates (6 samples per group). **p <0.05 *p<0.01*; 50, 100 and 500 = nicotine concentration of the exposure medium (ng/ml). The boxplots represent the 25th and 75th percentiles, median value is indicated by a line, minimum and maximum values by error bars and ° outliers, * extreme outliers.

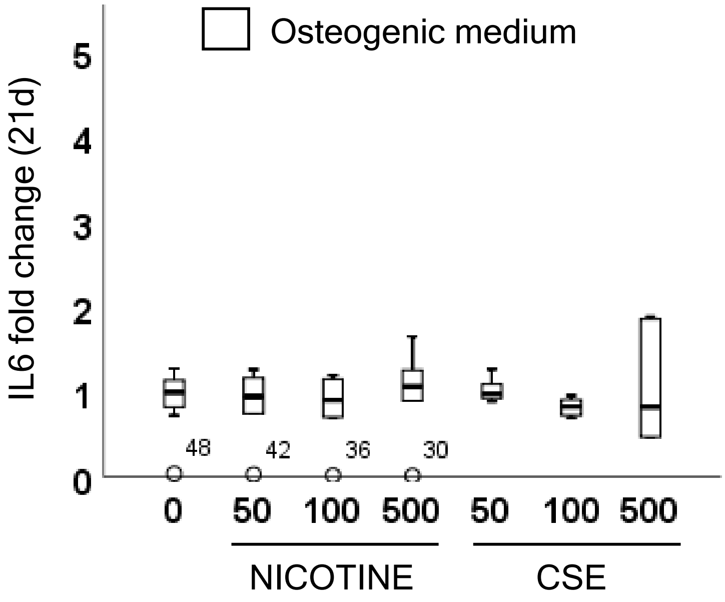

Supplementary figure 10. The effect of CSE and nicotine on cytokine secretion. The cytokine data (ELISA) of IL6 after 21 days of osteogenic differentiation. For the ELISA assay, results of the three cell lines normalized to respective controls are shown. Medium from three biological replicates were combined and the samples were measured as two technical replicates (6 samples per group). **p <0.05 *p<0.01*; 50, 100 and 500 = nicotine concentration of the exposure medium (ng/ml). The boxplots represent the 25th and 75th percentiles, median value is indicated by a line, minimum and maximum values by error bars and ° outliers, * extreme outliers.

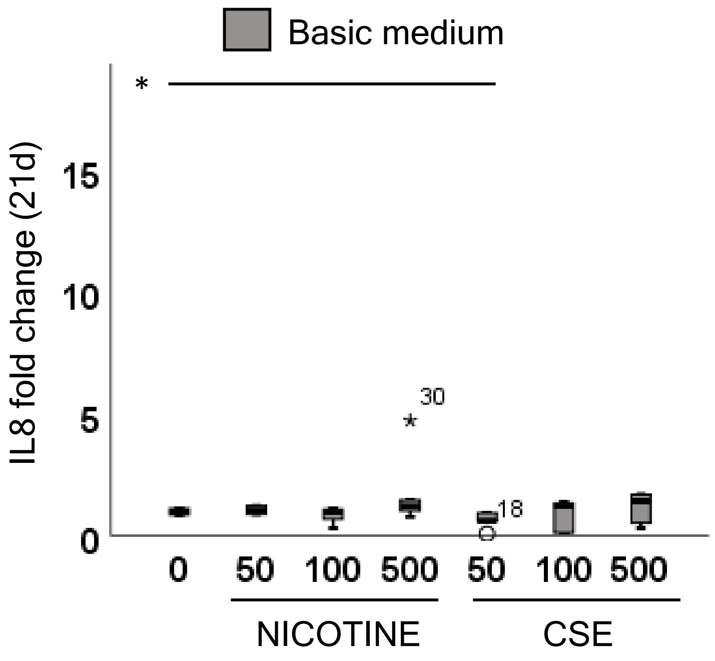

Supplementary figure 11. The effect of CSE and nicotine on cytokine secretion. The cytokine data (ELISA) of IL8 in the medium in basal condition was analysed after 21 days. For the ELISA assay, results of the three cell lines normalized to respective controls are shown. Medium from three biological replicates were combined and the samples were measured as two technical replicates (6 samples per group). **p <0.05 *p<0.01*; 50, 100 and 500 = nicotine concentration of the exposure medium (ng/ml). The boxplots represent the 25th and 75th percentiles, median value is indicated by a line, minimum and maximum values by error bars and ° outliers, * extreme outliers.

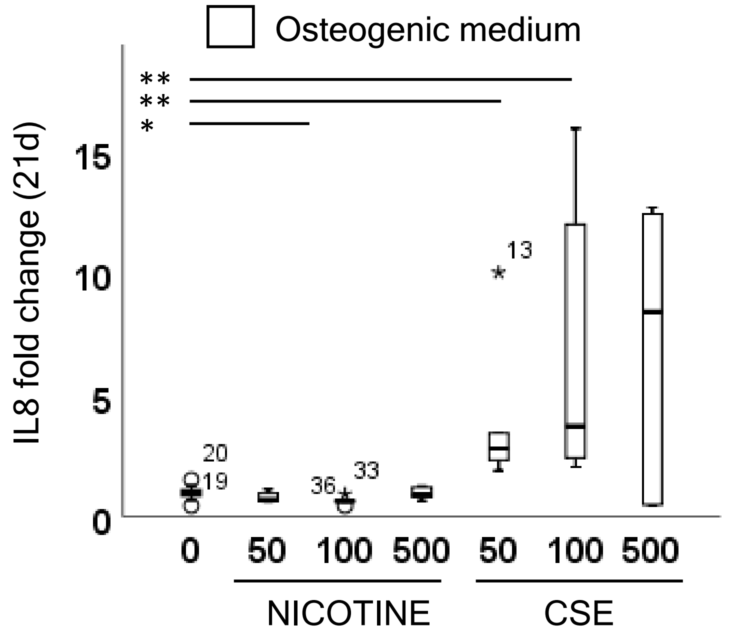

Supplementary figure 12. The effect of CSE and nicotine on cytokine secretion. The cytokine data (ELISA) of IL8 after 21 days of osteogenic differentiation. For the ELISA assay, results of the three cell lines normalized to respective controls are shown. Medium from three biological replicates were combined and the samples were measured as two technical replicates (6 samples per group). **p <0.05 *p<0.01*; 50, 100 and 500 = nicotine concentration of the exposure medium (ng/ml). The boxplots represent the 25th and 75th percentiles, median value is indicated by a line, minimum and maximum values by error bars and ° outliers, * extreme outliers.

© 2024 Heikkinen J. et al.
